# Inflammatory Bowel Disease: Clinical Diagnosis and Pharmaceutical Management

**DOI:** 10.18103/mra.v11i1.3135

**Published:** 2023-01-31

**Authors:** Amosy Ephreim M’Koma

**Affiliations:** 1. Department of Biochemistry, Cancer Biology, Neuroscience and Pharmacology, Meharry Medical College School of Medicine, Nashville, Tennessee, United States; 2. Department of Pathology, Anatomy and Cell Biology, Meharry Medical College School of Medicine, Nashville General Hospital, Nashville, Tennessee, United States; 3. Division of General Surgery, Section of Colon and Rectal Surgery, Vanderbilt University School of Medicine, Nashville, Tennessee, United States; 4. Affiliated Scientist Investigator, The American Society of Colon, and Rectal Surgeons (ASCRS), Arlington Heights, IL 60005, Unite States; 5. The American Gastroenterological Association (AGA), Bethesda, MD 20814, United States

**Keywords:** Inflammatory bowel disease, Ulcerative colitis, Crohn’s disease, Crohn’s colitis, Indeterminate colitis, Clinical diagnosis guideline, Molecular diagnostics, Medical Treatment guidelines, uneven representation of socioeconomic strata, inflammatory bowel disease care during COVID-19

## Abstract

Inflammatory bowel disease has an enormous impact on public health, medical systems, economies, and social conditions. Biologic therapy has ameliorated the treatment and clinical course of patients with inflammatory bowel disease. The efficacy and safety profiles of currently available therapies are still less that optimal in numerous ways, highlighting the requirement for new therapeutic targets. A bunch of new drug studies are underway in inflammatory bowel disease with promising results. This is an outlined guideline of clinical diagnosis and pharmaceutical therapy of inflammatory bowel disease. Outline delineates the overall recommendations on the modern principles of desirable practice to bolster the adoption of best implementations and exploration as well as inflammatory bowel disease patient, gastroenterologist, and other healthcare provider education. Inflammatory bowel disease encompasses Crohn’s disease and ulcerative colitis, the two unsolved medical inflammatory bowel disease-subtypes condition with no drug for cure. The signs and symptoms on first presentation relate to the anatomical localization and severity of the disease and less with the resulting diagnosis that can clinically and histologically be non-definitive to interpret and establish criteria, specifically in colonic inflammatory bowel disease when the establishment is inconclusive is classified as indeterminate colitis. Conservative pharmaceuticals and accessible avenues do not depend on the disease phenotype. The first line management is to manage symptoms and stabilize active disease; at the same time maintenance therapy is indicated. Nutrition and diet do not play a primary therapeutic role but is warranted as supportive care. There is need of special guideline that explore solution of groundwork gap in terms of access limitations to inflammatory bowel disease care, particularly in developing countries and the irregular representation of socioeconomic stratification with a strategic plan, for the unanswered questions and perspective for the future, especially during the surfaced global COVID-19 pandemic caused by coronavirus SARS-CoV2 impacting on both the patient’s psychological functioning and endoscopy services. Establishment of a global registry system and accumulated experiences have led to consensus for inflammatory bowel disease management under the COVID-19 pandemic. Painstakingly, the pandemic has influenced medical care systems for these patients. I briefly herein viewpoint summarize among other updates the telemedicine roles during the pandemic and how operationally inflammatory bowel disease centers managed patients and ensured quality of care. In conclusion: inflammatory bowel disease has become a global emergent disease. Serious medical errors are public health problem observed in developing nations i.e., to distinguish inflammatory bowel disease and infectious and parasitic diseases. Refractory inflammatory bowel disease is a still significant challenge in the management of patients with Crohn’s disease and ulcerative colitis. There are gaps in knowledge and future research directions on the recent newly registered pharmaceuticals. The main clinical outcomes for inflammatory bowel disease were maintained during the COVID-19 pandemic period.

## 1. Background

Inflammatory bowel disease (IBD) includes Crohn’s disease (CD) and ulcerative colitis (UC) are global emergent disease that are highly heterogeneous, debilitating, incurable, persistent, relapsing/ worsening, immune-arbitrated inflammatory pathologies of the digestive system canal [1]. UC causes inflammation and ulceration confined to the mucosal layer and the submucosae compartment of the colon and rectum [2]. CC compared to UC is segmental causing inflammation impacting the whole digestive system tract from the mouth to the anus. Further, CC cause inflammation deeper and can involve all the four colon layers that may engage other organs through fistulation [3,4].

Even though IBD that encompasses CD and UC, the two known digestive system pathologies have been considered to affect discrete of Western and Northern European ancestry, the epidemiology is changing dramatically with the steadily escalating incidence in hitherto low-incidence regions including resource-limited countries. The population of demographics of IBD in the United Kingdom, United States of America, Canada, and France are also changing, with increases in none-White born races and ethnicities [5–7]. It is consequently crucial to entirely apprehend the epidemiology and progression of IBD in discrete racial and ethnic groups, and the effects of race, culture, and ethnicity of access to care, use of resources, and disease related outcomes.

There are established guidelines for the diagnosis and pharmaceutical management of IBD [8,9] which includes international clinical practice tool recommendations that incorporates various best practices, and other evidences has widely been issued [10,11]. In the past two decades there have vastly been advances in research, i.e., molecular diagnostics and pharmacological evolution for IBD management [12,13]. The aim of this overview is to provide disease guidance consensus for healthcare professionals managing IBD, to ensure that investigation, diagnosis, treatment, and monitoring decisions are based on the best available common consent evidence, and to promote and ameliorate best accepted practice.

Etiobiopathogeneses of IBD are not yet fully elucidated of the opinion to be multifactorial [1,14,15] involving complex interaction linking the genetic, environmental and/or microbial factors and the immune responses [16]. The signaling pathway processes are arbitrated via intrinsic of the autoignition counter response to self-antigens [17–20].

Countries with strong economy i.e., US, Canada and Europe have continued to promote ambulatory regimens healthcare conveyance [21–24], third world countries have inadequate healthcare system services rendering limitations to getting the required quality of care [25] because of restricted capital and doctors and nurses luck knowledge because they are not trained about IBD [26]. In addition, for cost-effectiveness considerations and recommendations by the World Health Organization (WHO) and the World Gastroenterology Organization (WGO) [27,28], third world nations will wrestle in the inflexible economy. There is need of special guideline, currently not available that explore solution of infrastructure limitation in terms of access to IBD care in resource-limited countries and the inconsistency representation of socioeconomic stratification with a unique plan regarding how to surge this evolving pandemic [29]. The level of primary care and referral hospitals in third world nations face significant infrastructural insufficiencies and lack of regular clinical supervision and laboratory assessments and monitoring patients [30,29]. Patients face difficulty affording pharmaceuticals per the recommended and approval guidelines in the favor of wealthy countries.

## 2. Method

Information exploration guidelines regarding recommendation for the diagnosis and treatment of IBD was executed utilizing preestablished protocols in accordance with the quality of reporting meta-analyses of observational studies (MOOSE) [31, 32], MEDLINE and EMBASE were searched between 1980–2021. Further, Cumulative Index of Nursing and Allied Health Literature (CINAHL), PubMed, Google search engine, Cochrane Database and IBD-associated society organizations i.e. American Society for Gastrointestinal Endoscopy (ASGE), American College of Gastroenterology (ACG), British Society of Gastroenterology (BSG), International Foundation for Gastrointestinal Disorders (IFGD), American Gastroenterological Association (AGA), American Society of Colon and Rectal Surgeons (ASCRS), Society of American Gastrointestinal and Endoscopic Surgeons (SAGE), World Health Organization (WHO), United States Food and Drug Administration (USFDA), International Organization for the Study of Inflammatory Bowel Disease (IOIBD),European Medicines Agency (EMA), European Crohn’s and Colitis (ECC), American Crohn’s and Colitis (CCFA), CAG=Canadian Association of Gastroenterology (CAG) were also used.

## 3. Clinical Diagnosis

Standardized diagnostic test for IBD do not exist to date [33,34]. The common state-of-art-diagnosis of IBD relies on accumulation of clinical, radiologic, endoscopic, and histopathologic interpretation/ classification [35, 36]. This imprecise anthology is ambiguous in up to 15% of colonic IBD patients and are labeled as ‘‘indeterminate colitis’’ (IC) because the explication benchmark for CC and UC are unspecified [37,38]. There is another 15% of the colitides cases that are offered pouch surgery i.e., restorative proctocolectomy with ileal pouch-anal anastomosis (RPC-IPAA) for their ultimate UC diagnosis will have their initial UC diagnosis changed to ileal CD based on the subsequent clinical and histopathology changes indicate development of CD in the ileal pouch an indication that authentic CC was not diagnosed prior to colectomy [39,40]. Fifty percent of these patients with subsequent pouch CD will have their pouch extirpated or diverted [41,42].

## 4. Crohn’s disease

Crohn’s disease is diagnosed in at least four patients per 100000 live births in the United States and Canada. The incidence and prevalence is evolving internationally [29,43,44] specifically in the third world nations [30,29].. Clinically, CD differs from UC in that it may result in inflammation involving all the four intestinal walls i.e., mucosa, submucosa, muscularis and serosa and can also transpire in any parts of the digestive system tract, from the mouth, esophagus, stomach, duodenum, small intestine, colon, and rectum [36]. Further, CD may also penetrate to other systemic organs outside the GI tract through fistulation [3,4,45]. The clinical features for diagnosing CD includes an imprecise mixture of categorization system mentioned in section three above of IBD clinical diagnosis, and histopathological interpretation showing important, transmural, or granulomatous, asymmetric features [46,47]. Computed tomography (CT) enterography of the abdomen is the leading recommended and preferred first-line radiologic test used in the evaluation and/or assessment of CD. The diagnostic accuracy of magnetic resonance enterography / enteroclysis is equivalent to that of CT scans and prevents liability exposure to ionizing radiation. Endoscopic score metrics are the benchmark tool used to estimate the CD activity and often are used in the clinical setting trials to compute proof of the efficacy and safety of various drugs on causing and continuing remission and epithelial convalescence. There are many recommended scoring systems in the guideline but the most commonly used to measure clinical disease severity (CDS) include short inflammatory bowel disease questionnaire (SIBDQ, HBI- Harvey-Bradshaw index (HBI), and Lehmann score and Crohn’s Disease Activity Index (CDAI) [46,47].

## 5. Ulcerative Colitis

Most UC onset is in early adolescence [48]. Unmanaged UC results in chronic inflammation and ulcerations in the epithelial and submucosal layers restricted to the colon and rectum [36,48]. Approximately 15% of the patients may encounter toxic fulminant UC that may be admitted to hospital as emergence [48,49]. Establishing the UC diagnosis and disease state of a patient sample gastrointestinal pathologists depend on microscopic visual inspection and interpretation of distinct and/or stained tissue sections [50,51]. These performances provide with a compelling degree of discussion [52] and are abundance with exceptions [52,53]. Tutoring in pathology subspecialties is needed to improve and achieve the standard of care to avoid diagnostic ambiguity and delay [54,55]. Regardless of these illustrious benchmarks, ineradicable positions appear in which dispassionately cannot be conventionally dependable and where remarkable discrepancy of opinion occurs not to mention expert specialists [56]. Fundamental guideline overview and the consortium specialized review, disease activities of UC are summarized based on the Witts criteria and Truelove and Mayo Clinic score [57,58]. Mayo Clinic scores of 6–12 with an endoscopic subscore of 2 or 3 are aforethought moderate to severe disease. This guideline is explicated as hospital admitted patients with the following Truelove and Witts criteria: ≥6 hematochezia (bloody diarrhea) movements/day with at least 1 marker of inseparable toxicity, including heart beat/rate >90 beats/min, body temperature >37.8°C, blood hemoglobin <10.5 g/dL, and/or ESR (erythrocyte sedimentation rate) −30 mm/h [58].

## 6. Indeterminate Colitis

An estimated 15% of patients with colonic IBD cannot be delineated, especially during early stage of the disease and are termed ‘‘indeterminate colitis’’ (IC) due to non-definitive foundation of benchmark for UC and CC confounding effective treatment regimens and appropriate surgeries [37,59]. Elucidation of molecular biomolecules and different cellular signaling pathway mechanisms driving IBD heterogeneity is target to the future pharmaceutical inhibitor development to advancing patient care and quality of life [60–63]. Here, the central medical challenge is the discrimination of colonic IBD into the specific subtypes with accuracy. There are reports showing that human alpha defensin 5 (*DEFA5*, alias HD5) in the colon crypt mucosa with aberrant expression of Paneth cell-like cells (PCLCs) in areas identified with an ectopic colonic ileal metaplasia that is diagnostic of CC with a positive predictive value of 96% [64,65,103].

## 7. Clinical Presentation

The clinical presentation of IBD depends on the site and extent of mucosal inflammation, Quiscent, mild, moderate, and severe. The frequency of presenting symptoms is presented in Table 1 and in Table 2 are manifestations of IBD [164–172].

## 8. Molecular Diagnostic Advances in IBD

Molecular diagnostics research focuses on improving the diagnosis for patients with the predominantly colonic IBD subtypes of UC and CC with accuracy [36,66,67]. Current reported data indicate possible proteomic signatures that discriminate between UC and CC patients and predict the outcome of IC patients for their eventual progress to either authentic UC or CC [64,65]. Analytical evidence demonstrate that colonic apparent crypt cell-like cells (CCLCs) secreted human alpha defensin 5 (*DEFA5*, alias HD5) is aberrantly expressed in CC as compared to UC [66, 68]. Both the CCLCs and DEFA5 are not analogous of colon. Therefore presence of *DEFA5* in areas of the colonic mucosa with aberrant expansion of CCLCs identifies an area of ectopic colonic ileal metaplasia, positive for Paneth cell markers, that is consistent with the diagnosis of CC [68]. Detection of *DEFA5* in the biopsy tissues from the otherwise inexact diagnosed IC patients; and that UC patients who underwent RPC-IPAA surgery and later diagnosed with CD were more accurately differentiated from those which retained their original UC diagnosis by detection of *DEFA5* [39]. Together, this multipronged evidenced approach suggests that a *DEFA5* bioassay, can be adopted in the clinical practice to facilitate translational accessory for accurate IBD diagnosis, initial assessment prior to prescription of disease subtype appropriate pharmaceutical management and/or more importantly surgical options in case of conventional drug refractory.

## 9. Core Tip

To date there is no drug for cure for IBD. Optimized studies support the concept of the presence of multiple antibodies against enteric bacterial antigens in IBD [103]. Whether the production and releasing of antibodies is a result of barrier dysfunction induced by inflammation or a serologic finding secondary to IBD is discussible. Histopathology and clinical evaluation show that CD and UC, the two major classifications of IBD subtypes, are indeed discrete entities and have disparate causes and distinguishable mechanisms of tissue destruction/ damage and management [69,70]. The foundational inquiry is why the systemic innate immune process responds aggressively to indigenous innocuous, inextinguishable bacteria (the commensals), deliverance complex mixes of tissue by-product signatures (cytokines, chemokine, growth factors) and other substances that cause inflammation (antibody–antigen reaction against mucosal resistance). There has been a speculation that the trigger(s) of IBD may be identical, and the phenotype i.e., UC and CC are determined by the patient’s own immunity (level of strength) [16].

## 10. Treatment

**Figure 1.**
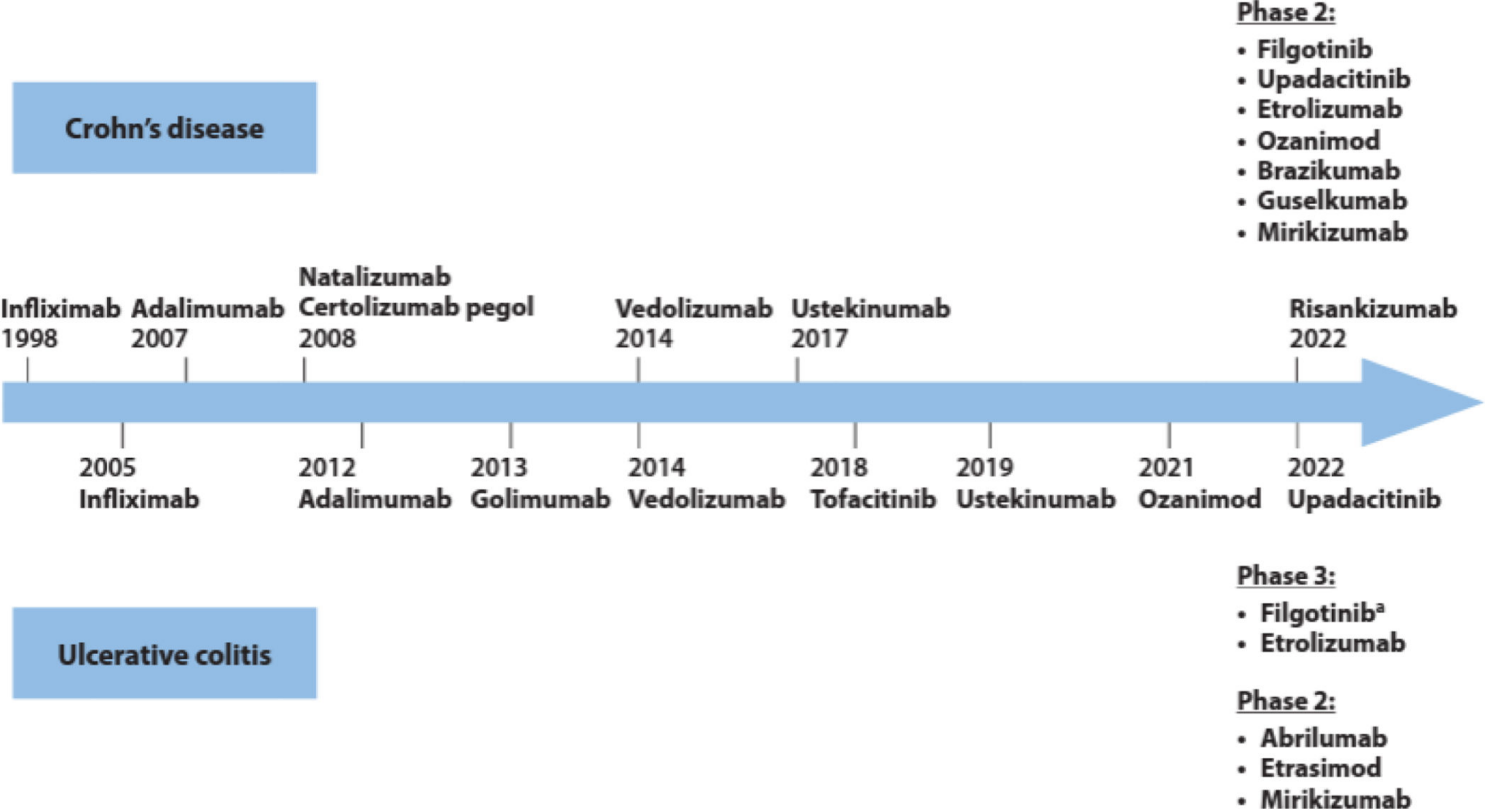
Timeline of drugs approved by the United States Food and Drug Administration for the management of IBD encompassing ulcerative colitis and Crohn’s disease, and published abstracts and articles for positive phase 2 and 3 clinical trials [163].

There are several international and individual national recommendation options in the treatment of IBD, of which some are specific for CD [8,71–73], UC specific [33,60,74–77] and/or both UC and CD [13, 78–80]. There are different recommended pharmaceutical classes for long-term treatment of moderate to severe IBD prescribed for promotion of remission and for prolonged maintenance remedy if they are effectual efficacious [77]. This specialized clinical practice is regarded professional and/or accepted by medical experts in this guideline and it is assumed that if a drug (excluding corticosteroids and cyclosporine) is initiated for and is efficacious for resulting of remission or response, it will be continued for maintenance of mucosal healing and remission [77]. There has been encouraging timeline of drugs approved by the United States Food and Drug Administration for the management of IBD, and published abstracts and articles for positive phase 2 and 3 clinical trials, Figure 1.

## 11. Pharmacological Treatment

The guideline by the American Gastroenterological Association (AGA) Institute’s Clinical Guidelines Committee approved by the AGA Governing Board is supported by a practical review that advises extended synthesis of the evidence-based from which these propositions were worked out [81]. Pharmacologically, IBD has no known cure to date [1]. The purpose of conservative clinical management of IBD is to induce and maintain remission in patients with dynamic/active disease [82,83]. Treatment strategies consist of symptomatic conservative pharmacological therapy and inevitably surgery depending on disease location, severity, and patients’ treatment history [84]. The conventional step-up approach consists of first-line therapy with classical treatment approaches such as aminosalicylates, corticosteroids, and immunomodulators (e.g., azathiopurine, 6-mercaptopurine) [85]. Due to variability in cellular processes that underlie the natural history of CC making diagnosis difficult, delayed, and appropriate treatment is often confounded [1,86]. In evaluating the best evidenced practice for optimizing treatment of IBD, the convergence is on anti-inflammatory drugs and therapies that restrain the immune system. The intestinal luminal innate immune response continues to be the important key focus of drug development and therapies [87,88]. The concept of dysbiosis of the anti-inflammatory, immune-, and microbiome-modulating has surfaced as a potential pathogenetic focus in IBD with a burgeoning interest in influencing the microbiome as a means of managing the disease in the therapeutic armamentarium [89,90]. Synchronous evolution of our understanding of the basic biology of IBD and triggers, there is an increasing appreciation for disconnect between patients’ symptomatology and IBD. As clinical trials have concurrently addressed both symptom scores and mucosal healing, physician scientists have gained a wide range appreciation for the fact that several symptoms may not be caused by active inflammation, and therefore focusing only on immunomodulatory therapies would not serve patients’ needs adequately. Furthermore, there is an emerging understanding of the significance of stress and psychological health in symptom experience and required therapy. The available conservative pharmacological treatment greatly reduces signs and symptoms, of which otherwise can be life threatening and fatal, bring about long-term remission [91,92]. With the correct diagnosis and treatment, people with the IBD can manage symptoms and improve their health quality of life (HQoL) [93,94].

Cytokines, chemokines, and growth factors are engaged in luminal intestinal homeostasis and pathological processes related triggers with IBD. The biological effects of secretagogues including several involved in the pathology of CD and UC, occur because of receptor-mediated signaling via the Janus kinase (JAK) and signal transducer and activator of transcription (STAT) DNA-binding families of proteins [95, 96].

More recently, newer biologics, cell-based therapies have emerged and been introduced, raining new aspirations that outcomes can be ameliorated. Despite this, in refractory cases, many patients are recommended a proctocolectomy requiring a stoma [12,41,97,98]. Since there is no drug for sure for IBD, the goal of IBD treatment is to reduce the inflammation that causes patient symptoms. In the best cases, this may lead not only to symptom relief but also to long-term remission, lower risks of adverse complexities and improved patient quality of life. Medical therapy has advanced dramatically in the last ten years with the establishment of targeted biologic therapies, the upsurge of older treatments, including rugs such as immunomodulators and 5-aminosalicylic acid (5-ASA), and a better understanding of the mucosal immune system and the genetics involved in the etiopathogenesis triggers of IBD. The therapeutic paradigm involves a step-up approach, moving to contentious powerful therapies only when milder therapies with fewer potential adverse side effects fail or when patients declare themselves to have an intrusive disease.

### Anti-inflammatory pharmaceuticals:

Anti-inflammatory pharmaceuticals are often the first step in the line of treatment of IBD. Anti-inflammatories include corticosteroids and aminosalicylates, such as mesalamine (Asacol HD, Delzicol, others), balsalazide (Colazal) and olsalazine (Dipentum). Which medication indicated depends on the anatomical area of gastrointestinal tract (GI) that is impacted.

### Immune system suppressors:

These medicines work in diverse ways to minimize the aggressive immune response that releases inflammation-inducing chemicals into the system. When released, these chemicals create an anti-body-antigen reaction against the mucosal resistance sequence of the patient that can damage the luminal inner lining of the digestive tract. Some examples of immunosuppressant drugs commonly used include azathioprine (Azasan, Imuran), mercaptopurine (Purinethol, Purixan) and methotrexate (Trexall).

### Biologics:

In the past twenty years, biological care with monoclonal antibodies targeting TNFα has become a groundwork of managing patients with IBD. Biologics are used routinely and are meant to overcome the limitations of therapies and ensure that individual patients can be treated with optimal drugs that are safe and precisely target IBD [99,100]. Pharmaceutic drug monitoring to guide the use of biologic therapy has widely been reported in AGA guidelines [101,102]. The guideline is predetermined for the multipurpose use of gastroenterology healthcare providers, primary care providers, surgeons, patients, and policymakers. The regulatory pathway of a biologic drug needs flourishing clinical trials that substantiate clinical efficacy as well as approval from regulatory agencies such as the United States Food and Drug Administration (FDA) and European Medicines Agency (EMA) [103,104]. Biotherapeutics, or biologics, are drug products fabricated using living instrumentation design systems. Their production typically involves genetically modified/ engineered animal, plant, or bacterial cells. Since their introduction, biologics have had a substantial impact on the clinical management of systemic inflammatory conditions such as IBD [105,106]. Biologics are a newer category of therapy in which treatment is directed toward mitigation or neutralizing chemicals in the body that are inflammation triggers. Examples include infliximab (Remicade), adalimumab (Humira), golimumab (Simponi), certolizumab (Cimzia), vedolizumab (Entyvio) and ustekinumab (Stelara).

### Biosimilars:

These are biological drugs like but different to the original biological agent called “originator biologic.” The advent of monoclonal antibodies, anti-TNF-α agents, has dramatically improved the therapeutic approach to patients with immune-mediated IBD [107]. These includes infliximab, adalimumab, and certolizumab in CD and infliximab, adalimumab, and golimumab which are effective and safe therapies to induce disease remission, achieve mucosal healing and improve UC patients’ HQoL [108,109]. Biosimilars represent a great opportunity to decrease health care costs and increase the access to effective medications to a larger number of patients particularly to those who are struggling economically is great advantage [3]. The bioequivalence of biosimilars is based on robust preclinical study evidence, but the clinical data supporting their efficaciousness and safeness in IBD mostly come from single-arm, noncontrolled cohort studies [110,111]. However, these molecules result are costly leading in high direct costs for the health care system, particularly in the long term [29]. A biosimilar is a biological brand that is highly like risk management plan (RMP), with indistinguishable clinically revealing disparities in terms of efficacy, safety, and purity [112,113]. Biosimilars and generic drugs both represent pursuit of a prize towards brand-name/trademark drugs. Even though a developed generic is an exact copy similar of the original small-molecule medicine, it is not feasible to manufacture identical copies of a biologic [114,115]. Since biologics are difficult to replicate, biosimilars are developed using alternate approaches such that the final product is almost identical to the RMP with respect to the initial amino acid sequence [116,117]. Due to the inherent variability of the living bacteria-based systems used in the development of biosimilar drugs, there is microheterogeneity between biosimilar and RMP [118].

In the mise-en-scène of biosimilars, regulatory agencies only need to make sure that high similarity and/or comparability is substantiated between the biosimilar and its RMP before a biosimilar candidate can officially be sanctioned and marketed, resulting in a simpler approval pathway [119]. The anticipation with biosimilars is that their entry into the market drives competition between drug developing companies, to compete and reduce prices comparably to how the generic market has, and to increase overall patient access to appropriate biologic treatments in the long-term. Currently, only two biosimilars have been approved for use in IBD in the United States: infliximab-dyyb and adalimumab-atto [120,121], Table 3. However, several anti-TNF biosimilars development have either been posed, are being evaluated in final-stage clinical trials, or are awaiting official approval from federal regulatory agencies i.e., The U.S. Food and Drug Administration (FDA).

### Antibiotics:

All kinds of antibiotics may be used in addition to other medications or when infection is a concern, especially in cases of perianal CD. Commonly prescribed antibiotics include ciprofloxacin (Cipro xr, Cetraxal, Otiprio) and metronidazole (Flagyl).

### Other medications and supplements:

In addition to controlling inflammation, some medications may be indicated to alleviate symptoms depending on the disease activity and severity of IBD. In medical practice, one or more of the following are in addition commonly prescribed:

### Anti-diarrheal medications:

A fiber supplement such as psyllium powder (Metamucil) or methylcellulose (Citrucel) to assuage mild to moderate diarrhea. For more-severe diarrhea, loperamide (Imodium A-D) may be efficacious alternative.

### Analgesics:

There are several pain reliever meds recommended for mild pain, i.e., acetaminophen (Tylenol, others). However, ibuprofen (Advil, Motrin IB, & others), naproxen sodium (Aleve) and diclofenac sodium likely will provoke the disease and worsen making symptoms become severe.

### Vitamins and supplements:

Vitamin deficiencies are common in patients suffering from IBD and is considered a potential pathogenic factor in their development [131,132]. Patients with IBD should be monitored and compensated for nutritional deficiencies [131–135].

### Dietary Therapy in IBD:

Based on science, both epidemiological studies and experimental studies there is no doubt that there are numerous associations that link IBD and diet [136–138]. Diet has partly been realized to contribute and play a role in the pathogenesis in inflammation, with research advances showing the effect of dietary exposures on the intestinal microbiome as well as mucosal integrity [139,140]. Specifically, the culture in industrialization of food and westernization of dietary practices such as fast food is suspected to play a key role in increasing incidence of IBD [141,142]. The use and ability of dietary intervention has shown to be beneficial to reduce inflammation as is illustrated in the efficacy of exclusive enteral nutrition (EEN) to induce remission in IBD, specifically in CD [143,144]. EEN, which is a balanced nutritionally complete liquid formula with the exclusion of all solid contents. Outlining areas of health coaching and dietary treatment options, the current knowledge about patient inspirations for pursuing dietary therapy and improvement for the decision-making process are emerging [145, 146]. When weight loss is severe enteral or parenteral nutrition is indicated to treat IBD. This will ameliorate generic nutrition and allow the bowel to rest. Bowel rest can slow inflammation in the short term. About 60% of patients with CC develop strictures, containing various degree of inflammation, fibrosis, and hyperplasia formation bowel obstruction [147]. Inflammatory strictures may benefit from the complementary use of pharmacologic anti-inflammatory and nutritional treatment to attain histologic and clinical remission. In a stenosis or stricture in the bowel, low-residue diet. This will help to minimize the chance that undigested food will get stuck in the luminal stricture part of the bowel continuity and lead to obstruction.

### Costs in IBD Management:

The economic implications of IBD are enormous [84]. Hospital admission rates and costs for IBD show an increasing trend assessed by specific pharmaceutical and disease features [148, 149]. In the US alone, the estimated annual direct treatment costs are greater than $6.8 billion, and indirect costs amount to an additional $5.5 billion [150,151]. Infliximab (Remicade) and adalimumab (Humira) introduction and maintenance therapies were cost-effective in comparison to standard care in patients suffering from moderate or severe CD; however, in patients with conventional-drug refractory CD, fistulizing CD and for maintenance of surgically induced remission incremental cost-effectiveness ratio (ICERs) were above acceptable cost-effectiveness thresholds [29,152]. In mild UC, induction of remission using high dose mesalazine (5-aminosalicylic acid (5-ASA)) is reported dominant in comparison to standard dose. In UC refractory to conventional evidence-based practice treatments, prescribing infliximab and adalimumab induction and maintenance treatment were not economically sound as compared to recommended standard care; however, ICERs for treatment with vedolizumab (Entyvio) and pouch surgery, RPC-IPAA are favorable to date. While biologic agents significantly ameliorate outcomes, they sustained significant inflated costs and hence are not economical, particularly for use as maintenance therapy [29,152]. The worthwhileness of biologic drugs may refine as market prices fall and with the introduction of biosimilars. Future research endeavors should focus on identifying optimal therapeutic strategies reflecting practical routine clinical practice, integrate indirect costs and evaluate estimates of lifespan costs and benefits.

### Management of Patients with IBD during the COVID-19 Pandemic

Coronavirus disease 2019 (COVID-19) pandemic has been a global tragedy that changed the traditional management plan of all diseases, with no exception of patients with IBD [11,153–155]. Bravely, the main clinical outcomes were maintained during the COVID-19 pandemic period largely because scheduled visits were replaced by phone calls and virtual consultations [156]. Virtual clinic follow-up using the contact center service (CCS) on the reorganization of a high-volume IBD centers and on the continuity of care during the COVID-19 outbreak. This approach could be implemented after the pandemic to optimize the resources of the IBD centers [156,157]. This has led to substantial changes causing interruption of non-essential endoscopic procedures and outpatient visits challenged, particularly on the assessment of disease activity by increasing the risk of relapse, disease complications, delays of new IBD diagnosis and detection of early post-operative recurrence of *de novo* CD [158]. The interventional performance of routine endoscopy was largely suspended in many IBD Clinic and Centers worldwide where undesirable acute respiratory syndrome coronavirus 2 (SARS-CoV-2) has spread [153]. Experts highlight different scenarios in which endoscopy should still be performed imperatively in unique circumstances in patients with IBD, as well as suggestion instructions regarding the use of personal protective equipment [153,159] for carry out safe procedures and the possible risks of postponing endoscopy in IBD and a post-pandemic plan for access to endoscopy as summarized by *Iacucci et al.* [153]. Clinical Practice Update (CPU) from expert emerge evidence that provide timely council about the treatment of patients with IBD during the CONVID-19. Admittedly, comments herewith provide perspective on a topic of high clinical importance that underwent internal peer review by the Clinical Practice Updates Committees and external peer review through standard procedures of Gastroenterology [11,153]. We are reminded however that as the understanding of the novel coronavirus progresses, IBD-specific issues and guidance may change beyond reasonable doubt [11,160]. Management of patient attending outpatient clinic with IBD in remission in the setting of asymptomatic SARS-CoV-2 infection or confirmed or suspected COVID-19 without systemic hyperinflammation syndrome are summarized in Tables 4, 5 and 6 [11,154,161,162].

## Conclusion:

IBD has become a global emergent disease. Refractory IBD is still a significant challenge in the management of patients with Crohn’s disease and ulcerative colitis. There are gaps in knowledge and future research directions on the recent newly registered pharmaceuticals. Serious medical errors are public health problem observed in developing nations around the world i.e., to distinguish IBD from bacterial or infectious parasitic diseases e.g., amoebiasis and shigellosis inadvertently contributes to severe delay in diagnosis and treatment. The main clinical outcomes for IBD were maintained during the COVID-19 pandemic period.

## Figures and Tables

**Table 1.** Frequency of presenting symptoms in IBD [164,165]

Symptom	CD (%)	UC (%)

Abdominal pain	62–95	33–76
Diarrhea	52–78	67–93
Weight loss	43–92	22–55
Hematochezia	14–60	52–97
Delayed growth	30–33	6
Fever	11–48	4–34
Perianal disease	25	0
Extraintestinal manifestation	15–25	2–16

**Table 2.** Manifestations of IBD [134,135,164–171]

System	Manifestation

Generalized	Fever
	Weight loss
	Malaise
	Anorexia
	Fatigue
	Nausea/ vomiting
Ocular	Uveitis
	Episcleritis
	Iritis
	Conjunctivitis
Oral	Cheilitis
	Stomatitis
	Aphthae
Pulmonary	Pulmonary vasculitis
	Fibrosing alveolitis
Vascular	Vasculitis
	Thrombosis
Hepatobiliary	Primary sclerosing Cholangitis
	Hepatitis
	Cholangitis
	Jaundice
Pancreatic	Pancreatitis
Gastrointestinal	Abdominal Pain
	Nausea/ vomiting
	Diarrhea
	Hematochezia
Renal/ Urinary	Nephrolithiasis
	Obstructive hydronephrosis
	Enterovesical fistula
	UTI
	Amyloidosis
Hematologic	Iron deficiency anemia Anemia of chronic disease Thrombocytosis Vitamin B_12_ deficiency Autoimmune hemolytic anemia
Endocrine	Decreased growth velocity Delayed sexual maturation
Integumentary	Erythema nodosum Pyoderma gangrenosum Perianal disease
Musculoskeletal	Osteopenia and osteoporosis Arthritis / Arthralgias Ankylosing spondylitis

**Table 3.** Clinical studies on infliximab-dyyb induction in inflammatory bowel disease.

Study	Population	Results	Definition of Outcome Response (Remission	Reference
Comparison Randomized 8 *vs.* 12 wks IFX maintenance	CD = 103	15 Point decrease in PCDAI **Remission**: PCDAI ≤10	8wks group: 56% 12 wks group: 24% (*p = 0.001*)	Hyams *et al*., [122]

Comparison Randomized 10 *vs.* 60 wks IFX maintenance	CD = 40	**Remission**: PCDAI <5	10 wks group: 83% 60 wks group: 61%	Ruemmele *et al*., [123]

Comparison Randomized 8 *vs.* 12 wks IFX maintenance	UC = 60	**Response**: Decreased in Mayo score By ≥ 30% and ≥3 points **Clinical remission**: Mayo score ≤2 with no individual subscore >1 and PUCAI <10	8wks group: 38% 12 wks group: 18% (*p = 0.146*) 54 wks group: 28.6%	Hyams *et al*., [124]

Comparison randomized high dose ADA (40 mg or 20 mg/body weight ≥40kg or 40 kg; n = 95 or Low dose (20 mg or 10 mg/body weight ≥40 kg or 40 kg; n = 95)	Moderate to severe CD = 188	**Response**: Decreased in PCDAI ≥15	High dose: 59% Low dose: 48% (*p = 0.073*)	Hyams *et al*., [125]
			High dose: 39% Low dose: 28% (*p = 0.075*)	
		**Remission**: PCDAI ≤10	High dose: 42% Low dose: 28% (*p = 0.075*)	

Prospective observational	CD = 46 UC = 32	Clinical remission rates at week 14 79% (CD), 59% (UC) Significant decrease in CRP, calprotectin	No adverse events reported	Jahnsen *et al*., [126]

Prospective multicenter	CD = 32 UC = 42	Clinical response at week 54 87.5% (CD), 100% (UC) Clinical remission rates at week 54 75% (CD), 50% (UC)	Adverse events in 11% of UC pts	Jung *et a*l., [127]

Prospective multicenter, national cohort	CD = 126 UC = 84	Clinical response at week 14 81.4% (CD), 77% (UC) Clinical remission rates at week 14 53.6% (CD), 58.6% (UC)	Adverse events in 17.1% in all pts	Gecse *et al*., [128]

Switch from RPM to Infleximab-dyyb	Pediatric CD = 32 UC = 7	Clinical remission rates 88% (CD), 57% (UC) Decreased in PCDAI, CRP, ESR	No adverse events reported	Sieczkowska *et al*., [129]

Prospective observational, Cohort switch	CD = 57 UC = 26	Clinical response at week 16 Calprotectin	No adverse events reported	Smits *et al*., [130]

**Abbreviations:** IBD: Inflammatory bowel disease. CD: Crohn’s disease. UC: Ulcerative colitis. CRP: C-reactive protein. IFX: Infliximab. ADA: Adalimumab. PCDAI: Pediatric Crohn’s Disease Activity. PUCAI: Pediatric Ulcerative colitis Activity Index. WKS: Weeks. DAI: Disease Activity Index. ESR: Erythrocyte sedimentation rate. HBI: Harvey Bradshaw Index. OCDAL: Pediatric Crohn’s Disease Activity Index.

**Table 4.** Management of patients attending outpatient clinic with quiescent inflammatory bowel disease in the scenario of asymptomatic severe acute respiratory syndrome coronavirus 2 infection or confirmed or suspected coronavirus disease 2019 [11, 154,161,162].

Management	
Asymptomatic infection with Taper or SARS-CoV-2	(1) Budesonide, aminosalycilates, antibiotics, and topical therapy may be maintained; (2) Hold immunomodulators tofacitinib, and biologics for 2 wk; (3) withdraw systemic corticosteroids (prednisone); and (4) Monitoring for 2 wk for COVID-19 symptoms.
Mild COVID-19	(1) Budesonide, aminosalycilates, antibiotics, and topical therapy may be maintained; (2) Hold immunomodulators, tofacitinib, and biologics for 2 wk; and (3) Taper or withdraw systemic corticosteroids (prednisone)
COVID-19 with pulmonary immune-involvement without SHS	(1) Budesonide, aminosalycilates, antibiotics, and topical therapy may be maintained; (2) Hold immunomodulators, tofacitinib, and biologics for 2 wk; and (3) Taper or discontinue systemic corticosteroids

Immunomodulators refer to thiopurines and methotrexate. SARS-CoV-2: severe acute respiratory syndrome coronavirus 2; COVID-19: Coronavirus disease 2019; SHS: Systemic hyperinflammation syndrome.

**Table 5.** Management of patients attending outpatient clinic with mildly active inflammatory bowel disease in the scenario of the asymptomatic severe acute respiratory syndrome coronavirus 2 infection or confirmed or suspected coronavirus disease 2019 [11,15,4,161,162].

	Management
Asymptomatic infection or with SARS-CoV-2	(1) Budesonide, aminosalycilates, antibiotics, and topical therapy may be used if needed; (2) Holds immunomodulators, tofacitinib, and biologics for 2 wk; (3) Taper withdraw corticosteroids (prednisone < 20 mg/d); and (4) Monitoring for 2 wk for COVID-19 to present.
Mild COVID-19	(1) Budesonide, aminosalycilates, antibiotics, and topical therapy may be used if needed; (2) Hold immunomodulators, tofacitinib, and biologics for 2 wk; (3) Taper or withdraw systemic corticosteroids); and (4) Monitoring for 2 wk for COVID-19 symptoms to disappear.
COVID-19 with pulmonary unit involvement without SHS	(1) Budesonide, aminosalycilates, antibiotics, and topical therapy may be used if necessary; (2) Hold immunomodulators, tofacitinib, and biologics for at least 2 wk or COVID-19 resolves; and (3) Taper or withdraw systemic corticosteroids.

**Table 6.** Management of patients attending outpatient clinic with moderately to severely active inflammatory bowel disease in the scenario of asymptomatic severe acute respiratory syndrome coronavirus 2 infection or confirmed or suspected coronavirus disease 2019 [11, 154,161,162].

	Management
Asymptomatic infection necessary with SARS-CoV-2	1) Restrict the use of prednisone ≤ 40 mg/d if necessary; (2) Avoid immunomodulators and to facitinib; with SARS-CoV-2 (3) Escalate to biologics as (preferably in monotherapy); and (4) Thromboprophylaxis
Mild COVID-19	1) Restrict the use of prednisone ≤ 40 mg/d if necessary; (2) Avoid starting or stopping, if in use, immunomodulators, and tofacitinib; (3) Escalate to biologics and dose optimization as necessary (preferably in monotherapy); and (4) Thromboprophylaxis
COVID-19 with pulmonary Escalate involvement without SHS with infectious	(1) Restrict the use of prednisone ≤ 40 mg/d if necessary; (2) Avoid starting or stopping pulmonary involvement immunomodulators, and tofacitinib; (3) to biologics and dose optimization as without SHS necessary (preferably) in monotherapy based on balance of benefits and risks; consultation diseases expert for possible COVID-19 treatment with antiviral or experimental anticitokine therapy; and (4) Thromboprophylaxis

Immunomodulators refer to thiopurines and methotrexate. SARS-CoV-2: severe acute respiratory syndrome coronavirus 2; COVID-19: Coronavirus disease 2019; SHS: Systemic hyperinflammation syndrome
